# Supplementary material for: Implementation of Cellulose-Based Filtration Aids in Industrial Sunflower Oil Dewaxing (Winterization): Process Monitoring, Prediction, and Optimization
Source: Foods. 2024 Sep 19;13(18):2960. doi: 10.3390/foods13182960 (PMC11431447; doi:10.3390/foods13182960)
Supplement: Supplementary file 1 [file foods-13-02960-s001.zip › foods-3124574-supplementary.pdf]

Supplementary material

Implementation of Cellulose-Based Filtration Aids in Industrial Sunflower Oil Dewaxing (Winterization): Process Monitoring, Prediction, and Optimization

Tanja Lužaić[1], Katarina Nedić Grujin[1,2], Lato Pezo[3], Branislava Nikolovski[1], Zoran Maksimović[4] and Ranko Romanić[1,*]

[1] Faculty of Technology Novi Sad, University of Novi Sad, Bulevar cara Lazara 1, 21000 Novi Sad, Serbia; tanja.luzaic@tf.uns.ac.rs (T.L.); katarina.nedic.grujin@gmail.com (K.N.G.); barjakb@uns.ac.rs (B.N.)
[2] Dijamant Ltd., Temišvarski drum 14, 23000 Zrenjanin, Serbia
[3] Institute of General and Physical Chemistry, University of Belgrade, Studentski trg 12/V, 11158 Belgrade, Serbia; latopezo@yahoo.co.uk
[4] Faculty of Pharmacy, University of Belgrade, Vojvode Stepe 450, 11221 Belgrade, Serbia; zmaksim1@pharmacy.bg.ac.rs
* Correspondence: rankor@uns.ac.rs; Tel.: +381-21-485-3700

Case study – economic justification of cellulose-based filtration aids usage

Brief overview of the prices of the filtration aids used in the 57 filtration cycles examined is shown:

- Conventional non-cellulosic filtration aid

Depending on the wax content of the oil being winterized, the price ranges from a minimum of 96.00 RSD/t of filtered oil (FO) to a maximum of 168.00 RSD/t FO, with an average price of 130.00 RSD/t FO for all 57 filtrations (1.00 euro = 118.00 RSD).

- Cellulose-based filtration aid FA-I

Depending on the wax content of the oil being winterized, the price ranges from a minimum of 40.19 RSD/t of filtered oil (FO) to a maximum of 282.10 RSD/t FO, with an average price of 89.29 RSD/t FO for all 57 filtrations.

- Cellulose-based filtration aid FA-II

Depending on the wax content of the oil being winterized, the price ranges from a minimum of 57.84 RSD/t of filtered oil (FO) to a maximum of 375.00 RSD/t FO, with an average price of 129.29 RSD/t FO for all 57 filtrations.

Annually, the production plant of the company Dijamant Ltd. in Zrenjanin produces about 57,000.00 t of winterized oil, using cellulose-based filtration aids. Approximately 300 t of filter cake are generated. The disposal costs for exhausted cellulose-based filtration aids are 0.00 euros, while the cake is sold, and the profit from this is not included in the calculation. If conventional non-cellulosic aid is used, the disposal cost amounts to 22,500.00 euros.

The maintenance costs of equipment when working with cellulose-based filtration aid on an annual basis amount to 67,600.00 euros, while when dealing with conventional non-cellulosic filtration aid, it amounts to 132,000.00 euros.

When discussing cost-efficient and economical filtration, it is important to note that compared to conventional non-cellulosic (mineral) filtration aids, the purchasing costs of cellulose are usually higher. This fact, however, is more than compensated for by the positive properties of cellulose based filtration aids. Its low wet cake density results in considerably less filtration aid being consumed than with mineral filtration aids. In addition, product losses are reduced due to high permeability, and disposal of filter cake does not incur any costs. Moreover, using cellulose-based aids substantially reduces plant maintenance costs. To conclude, filtration with cellulose-based filtration aids is much more economical than with conventional filter aids. Due to all the aforementioned facts and the presented calculations, the company Dijamant Ltd. in Zrenjanin has decided to use this filtration aids, which is in operation in their production plant.

It is also important to emphasize that this data is only valid for this single production plant, the manner of equipment usage, the specific quality of oil being refined, working conditions, etc. If any of the aforementioned changes occur in this production plant, or if industrial winterization is performed in a different plant with different equipment, crude oil, etc., this data will not remain the same.
